# Acute nerve compression and the compound muscle action potential

**DOI:** 10.1186/1749-7221-3-1

**Published:** 2008-01-22

**Authors:** Mark M Stecker, Kelly Baylor, Yiumo Michael Chan

**Affiliations:** 1 Department of Neurology, Geisinger Medical Center, Danville, PA 17822 USA; 2 Weis Center for Research, Geisinger Medical Center, Danville, PA 17822 USA; 3 McColl-Lockwood Laboratory for Muscular Dystrophy Research, Department of Neurology, Carolinas Medical Center, PO Box 32861, Charlotte, NC 28232 USA

## Abstract

Detecting acute nerve compression using neurophysiologic studies is an important part of the practice of clinical intra-operative neurophysiology. The goal of this paper was to study the changes in the compound muscle action potential (CMAP) during acute mechanical compression. This is the type of injury most likely to occur during surgery. Thus, understanding the changes in the CMAP during this type of injury will be useful in the detection and prevention using intra-operative neurophysiologic monitoring.

The model involved compression of the hamster sciatic nerve over a region of 1.3 mm with pressures up to 2000 mmHg for times on the order of 3 minutes. In this model CMAP amplitude dropped to 50% of its baseline value when a pressure of roughly 1000 mmHg is applied while, at the same time, nerve conduction velocities decline by only 5%. The ability to detect statistically significant changes in the CMAP at low force levels using other descriptors of the CMAP including duration, latency variation, etc alone or in conjunction with amplitude and velocity measures was investigated. However, these other parameters did not allow for earlier detection of significant changes.

This study focused on a model in which nerve injury on a short time scale is purely mechanical in origin. It demonstrated that a pure compression injury produced large changes in CMAP amplitude prior to large changes in conduction velocity. On the other hand, ischemic and stretch injuries are associated with larger changes in conduction velocity for a given value of CMAP amplitude reduction.

## Background

Intra-operative neurophysiologic monitoring is an important clinical tool that provides surgeons with real time feedback on the integrity of critical neural structures enabling the surgeon to alter the surgical plan if there is a warning of impending neurologic injury [1,2]. One particular application involves stimulating a nerve proximally while continuously recording compound muscle action potentials (CMAP's) during a surgical procedure that places the nerve at risk. For this application, it is critical to understand the physiology of nerve injuries occurring over seconds to minutes. Although the interest in the physiology of hyperacute nerve injury is relatively new, there has been much study into the changes in peripheral nerve conduction during compression beginning with the pioneering studies of Erlanger and Gasser [3]. Despite these early physiologic studies, most prior work in the reaction of peripheral nerve to injury has been related to chronic or subacute injury either by imaging [4-7], clinical or chronic neurophysiology [8]. A few studies [9-12] beginning with Causey and Palmer [11] have investigated the neurophysiologic effects of compression over shorter time scales. Only one of the above studies has monitored the CMAP and evaluated changes over the period of seconds important for neurophysiologic monitoring [11]. In that particular study, however, only CMAP amplitudes were measured and not changes in the shape of the CMAP or its latency which are also critical components of neurophysiologic monitoring.

The goal of this paper was to study in detail the changes in the CMAP, reflecting the function of axons of alpha motor neurons, during hyperacute nerve injury. In particular, conduction velocities, CMAP amplitudes, CMAP duration, and the shape of the CMAP were all be studied as well as the presence of spontaneous electromyographic (EMG) activity.

## Methods

### Use of animals

Under a protocol approved by the Weis Research Center IACUC (#173-06) 16 sciatic nerves from 10 normal male golden Syrian (F1-B) hamsters were analyzed. Hamsters were purchased from BioBreeders (Watertown, MA). These hamsters have a relatively large body size and can withstand surgical procedures well. All studies were performed under pentobarbital anesthesia (90 mg/kg administered by intraperitoneal injection).

### Recording the CMAP

Recordings of the CMAP were made from the platinum subdermal needle electrodes (Model E2-48, Astro-Med, Inc., West Warwick, RI) placed in the muscles of the hind paw. The sciatic nerve was stimulated proximally at the spine using similar subdermal needle electrodes placed in tripolar fashion with 2 mm separation between the electrodes. Stimulation was accomplished with a Grass S88 stimulator connected to a Grass PSIU6 current isolation unit. Stimulation was increased in the range of 2–15 mA to assure supramaximal stimulation at the beginning of the experiment. The duration of each stimulus was chosen as 0.01 msec.

The signal from the recording electrodes was amplified by Grass Model 8 amplifiers (Astro-Med, Inc., West Warwick, RI) with the high frequency filter set at 10 kHz and the low frequency filter set at 0.3 Hz. The sensitivity was 300 *μ*V/mm. Continuous recordings of spontaneous muscle activity were amplified and directed to a loudspeaker so that spontaneous electromyographic activity could be documented. The signal was digitized using a PCI-6031E 64 channel, 16 bit, 100 kHz data acquisition card (National Instruments, Austin, TX). Stimulation was performed at a rate of 5/sec and the average of 20 traces was computed prior to saving the response. This number of averages was chosen as a compromise between the noise reduction associated with additional averaging and the problems of jitter related distortions in waveform and reduced temporal resolution for changes in CMAP characteristics associated with averaging. Thus, CMAP's were recorded every 4 seconds.

The recordings of the CMAP's were integrated with continuous measurements of the hamster's rectal temperature as well as the output of a Shipmo DFS-1 force gauge (Shimpo Instruments, Itasca, IL) with a measurement accuracy of 0.1 g. Software (Measurement Studio from National Instruments) was used to record annotations in synchrony with the CMAP recordings and enabled both manual and automatic marking of the CMAP's.

**Figure 1:**
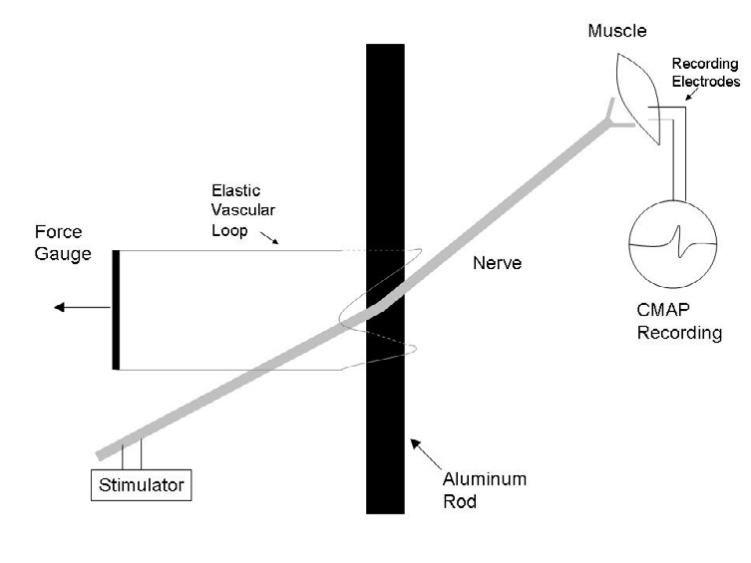
Basic setup for the nerve compression study.

After dissection of the sciatic nerve, a thin metal rod (1.5 mm diameter) was placed under the nerve and secured. Standard 1.3 mm wide vascular loops were wrapped around the nerve as shown in Figure 1 in order to cause compression of the nerve as tension was applied to the vascular loops. This scheme mimics some types of injury that might be seen during surgical procedures such as a clip being placed on a nerve, an instrument inadvertently pushing against a nerve or a nerve being trapped against another structure by a tie or suture.

The compressive force on the nerve increases gradually with the tension in the loops. This relationship was measured directly by using known weights to produce s specific tension in the vascular loops and then using the force gauge to measure the smallest force required to lift the vascular loop out of contact with the metal rod. A linear regression was applied to this data to obtain the approximate empirical relations:

(1) $$\begin{array}{l} F(\text{Newtons)}=0.004*T\text{(g)}\\ P\left(\text{mmHg}\right)=18.4*T(\text{g}) \end{array}$$

where T is the tension in the vascular loops as measured by the force gauge, F is the force on the nerve and P is the pressure on the nerve. It should be noted that the compressed section of nerve is exposed to atmospheric oxygen throughout the experiment and is unlikely to become ischemic

Before recording data, the stimulus intensity was adjusted to obtain a supramaximal stimulus and the recording and stimulating electrodes were adjusted to obtain a high amplitude (>500 *μ*V) response. The baseline condition began once all adjustments were complete and the responses were stable. This was followed by compression of the nerve corresponding to 20 g tension in the loops for up to 3 minutes (1^st ^compression). The compression was terminated prior to the 3 minute period if the amplitude of the CMAP declined more than 50%. This was followed by a 3 minute recovery period (1^st ^recovery) and compression to 80 g tension in the loops (2^nd ^compression). Again, compression was followed by a 3 minute recovery time (2^nd ^recovery). After this, compression on the nerve was again instituted and increased until the CMAP disappeared (3^rd ^compression). This was followed by a 3 minute recovery period (3^rd ^recovery). CMAP's were recorded continuously during the entire period

### Statistical analysis

The term latency always refers to the time delay between the stimulus and the onset of the CMAP and the term amplitude refers to the maximum peak to peak amplitude. Computation of conduction velocities assumed a synaptic delay of 0.5 msec [13]. All computed velocities were corrected to the values corresponding to 37°C according to the relation derived from an analysis of baseline latencies:

(2) Latency_corrected _= Latency*e^-.032*(37-*T*)^

where T is the rectal temperature at the time of the latency measurement and the corrected latency is that expected at 37°C.

Four other features of the CMAP are computed, the duration, the area under the curve (AUC), the mean value of the CMAP latency and its variance. The duration of the CMAP is measured as the difference between the time of the first and last noticeable deflection of the CMAP. Since the CMAP generally has components above and below baseline, the area under the curve is computed using Simpson's rule applied to the absolute value of the CMAP

(3) $$\text{AUC}=\int_{t_{start}}^{t_{stop}}\left|V(t)\right|dt$$

where t_start _is the shortest time after stimulation at which reliable data is available and t_stop _is the lastest time for which a CMAP is present. It is also possible to define the mean latency of the CMAP, *τ*, and its standard deviation, *τ*_*s*_, using the square of the CMAP amplitude as a weighting function:

(4) $$\tau =\frac{\int_{t_{start}}^{t_{stop}}V^{2}(t)tdt}{\int_{t_{start}}^{t_{stop}}V^{2}(t)dt}$$

(5) $$\tau_{s}=\sqrt{\frac{\int_{t_{start}}^{t_{stop}}V^{2}(t){\left(t-\tau \right)}^{2}dt}{\int_{t_{start}}^{t_{stop}}V^{2}(t)dt}}$$

In order to facilitate comparisons between the changes in the CMAP seen during different experiments, it can be useful to look at the relative variations in these CMAP descriptors. In order to do this, the mean value of the parameter during the baseline (pre-compression) state is computed and the relative values of the parameter throughout the remainder of the experiment are computed by dividing the actual value of the parameter by its mean value in the baseline state. Thus, the relative values of each CMAP parameter begins at 1.

Of interest from the neurophysiologic monitoring standpoint was a determination of the time at which the first statistically significant changes in one of the above discussed CMAP parameters occurred during the experiment. A simple method to determine this time involved performing a repeated measures ANOVA in the normalized variable under study starting with the first two stages of the experiment (after the baseline) and then adding successive stages to the ANOVA until a statistically significant effect is noted. This successive ANOVA is not the traditional approach but it accurately reflects the situation that occurs in neurophysiologic monitoring where all past data is used to determine if there has been a significant change up to the point in question. Because of the assumptions implicit in the ANOVA, similar computations were also carried out using the Friedman non-parametric ANOVA (Statistica, Tulsa, OK). The only difference between these two analyzes was that the baseline data from the normalized variables which could not be included in the parametric ANOVA because of the absence of variance were included in this analysis which was based on ranks. In addition, a t-test was used to compare the values of CMAP parameters when spontaneous EMG activity is recorded and when it is not. Spearman rank correlations and linear regression were used as appropriate to determine whether there were significant relations between continuous variables. Statistical significance was taken as p < .05.

## Results

**Figure 2:**
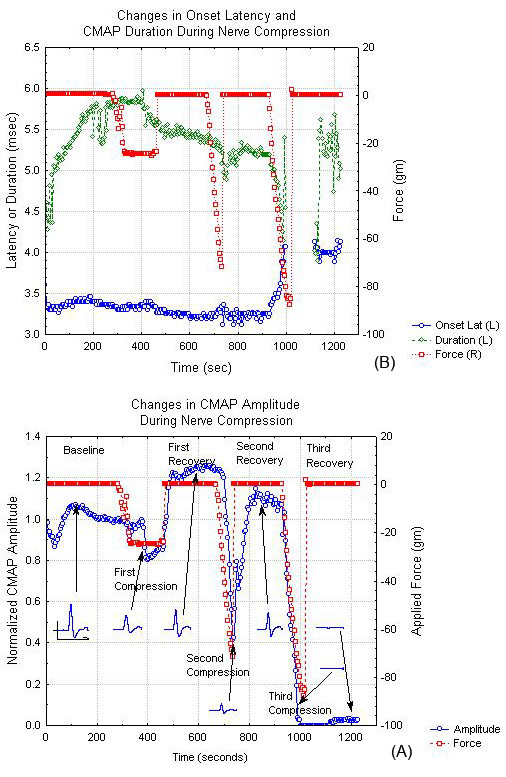
CMAP parameters and nerve compression tension as a function of time during a typical nerve compression experiment. (A) Changes in CMAP amplitude and CMAP waveform. (B) Changes in CMAP onset latency and duration. In both cases, the applied force is shown on the right y-axis and the CMAP parameter value on the left y-axis.

The typical changes in the CMAP during compression of a single nerve are shown in figure 2. Specifically, figure 2A shows changes in amplitude while figure 2B documents the corresponding changes in the CMAP onset latency and duration. This figure illustrates four common findings during hyperacute nerve compression. First, compression to 20 g of tension (P = 370 mmHg) leads to very little acute reduction in CMAP amplitude (~20%, figure 2A) while compression to 80 g (P = 1470 mmHg) causes significant CMAP amplitude reduction (~60%) but only minimal CMAP latency increases (figure 2B). Second, there is considerable variation in the CMAP duration but durations typically drop as the CMAP amplitude declines. Third, the CMAP amplitude during the recovery period may exceed that prior to compression. Fourth, at the high pressure levels (>80 g) used in this study, there are very rapid responses to compression.

**Figure 3:**
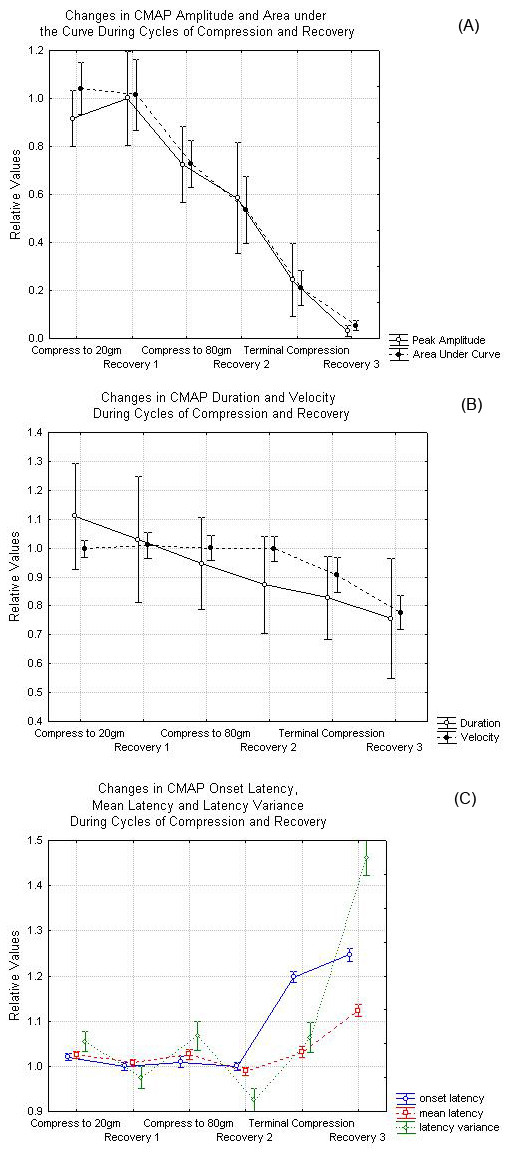
(A) Changes in amplitude and area under the curve at various points during the compression and recovery cycles. (B) Changes in nerve conduction velocity and CMAP duration during the various stages of nerve compression. (C) Changes in CMAP onset latency, mean latency and latency variance during nerve compression and recovery. The data in this figure represent averages over all nerves. In each case, the error bars represent 95% confidence intervals or roughly 2 standards errors of the mean above and below the central value.

Knowing these general properties, it is useful to look at the responses in the entire group of studied nerves. The CMAP amplitude is reduced by 50% for the first time during compression at a tension T = 52.7 g (std 22.7, min 18, max 79) corresponding to a mean pressure of 970 mmHg. The AUC for the CMAP is strongly correlated with the peak to peak amplitude. Linear regression of the normalized AUC on the normalized amplitude yields a slope of 0.95 (+/- .008) with R^2 ^= .74. As expected, the AUC drops to 50% at a tension T = 54.4 g (P = 1001 mmHg), similar to the tension at which the CMAP amplitude drops to 50% of baseline. Figure 3A shows the mean changes in amplitude and AUC during each phase of the experiment averaged over all nerves. The CMAP amplitude and AUC reductions first reach statistical significance during the phase in which the nerve is subjected to compression at a tension of 80 g. Figure 3B shows the mean changes in velocity and duration of the CMAP during cycles of compression and recovery. Nerve conduction velocity changes minimally until levels of compression significant enough to reduce the CMAP amplitude to less than 20% of its initial value are attained. Although small, the 5% reduction in conduction velocity seen during the 80 g compression is statistically significant. Figure 3B also confirms the decline in CMAP duration during compression. There is much variability in this measure and significant differences are not seen until the terminal compression phase. Figure 3C demonstrates that the CMAP onset and mean latency, do not change significantly during the early phases of compression. Significant change is only observed at the terminal compression phase. Although the latency variance does increase during compression and decrease during recovery, this effect does not reach statistical significance until the terminal compression phase. We also investigated whether any combination of the above parameters show statistically significant changes earlier in the experiment. These included products of the primary parameters discussed earlier in such combinations as amplitude*velocity, amplitude*velocity*duration, amplitude*velocity* *τ*_*s*_. However, none of these derived parameters showed statistically significant changes in the CMAP at an earlier point than either the CMAP amplitude or velocity alone. It should be noted that similar results were obtained using both the parametric and non-parametric ANOVA testing.

**Figure 4:**
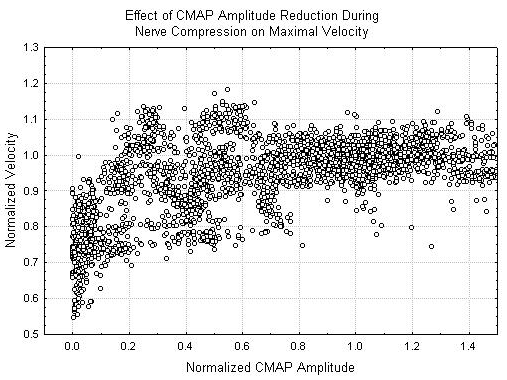
Changes in relative CMAP velocity as a function of relative CMAP amplitude. In each case, relative CMAP values are derived from the raw measured values of that parameter by dividing the raw values by the mean value of the given parameter in the baseline state.

**Figure 5:**
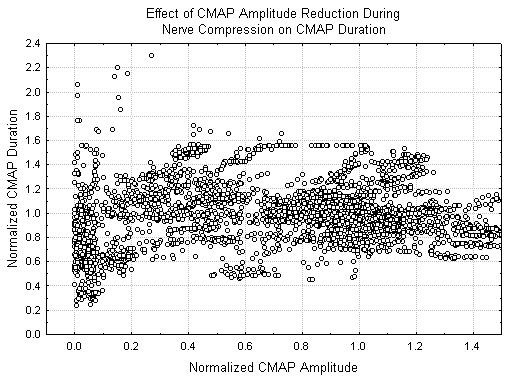
Changes in relative CMAP duration as a function of relative CMAP amplitude.

In all cases where a CMAP was recordable, the maximum velocity was never less than half of its initial value. In order to better elucidate the changes in the CMAP, figures 4 and 5 show the normalized CMAP velocity and CMAP duration respectively from each recorded potential as a function of the normalized CMAP amplitude. In both of these cases, no change in the measured parameters greater than 10% occurs until the CMAP amplitude is reduced by over 80%. Similar effects of the mean latency and latency variance are noted.

An examination of the behavior of individual nerves shows that the CMAP amplitude returns to baseline or above in 9/16 nerves in the recovery period after the 1^st ^compression (20 g) while 16/16 achieved amplitudes >30% of baseline. After the 2^nd ^(80 g) compression, only 4/16 nerves returned to baseline amplitude and 10/16 reached amplitudes greater than 30% of baseline. After the 3^rd ^compression, during which the tension was adjusted to make the CMAP disappear, 0/14 nerves achieved an amplitude >30% of baseline during the recovery period. Thus, the ability to recover is better after compressions that cause smaller declines in CMAP amplitude. Despite the minor changes seen in velocity, only 44% of nerves recovered to equal or better velocity after the 1^st ^compression at 20 g, 29% after the 2^nd ^compression at 80 g and 0% after the final compression.

In order to determine if the degree of CMAP amplitude reduction during low level compression predicted the degree of CMAP reduction with higher level compression, a Spearman rank correlations is performed. The degree of reduction in the CMAP amplitude during the 1^st ^compression to 20 g is positively and significantly correlated with the CMAP amplitude during the 2^nd ^compression to 80 g (Spearman R = 0.5, p < .05) However, there is no significant relation between the reduction in amplitude during the 1^st ^compression and the CMAP amplitude during recovery from 80 g compression (Spearman R = 0.275, p > .05). There is also no significant correlation between the CMAP amplitude reduction during the 1^st ^compression and CMAP amplitude reduction during the final recovery. The degree of reduction in velocity during the 1^st ^compression is positively correlated (Spearman R = 0.77, p < .01) with the degree of velocity reduction during the 2^nd ^compression but not with velocity during the recovery period. On the other hand, there are positive but statistically insignificant correlations between the changes in velocity during the 1^st ^compression to 20 g and the amplitude reductions during the 2^nd ^compression to 80 g.

Trains of spontaneous EMG activity are more commonly recorded when the CMAP amplitude is significantly reduced from baseline. The normalized CMAP amplitude when there was no spontaneous EMG activity was 0.657 while the mean amplitude was 0.36 when spontaneous EMG activity was seen (t = -2.65, p < .01, N = 3897). The CMAP duration was also shorter when spontaneous EMG was recorded (0.989 vs 0.82 p < .01 t = -2.65, N = 3897) than when it was not. There was no effect of the rate of CMAP change on the appearance of spontaneous EMG activity.

## Discussion

This study has demonstrated that the uniform response to mild to moderate hyperacute nerve compression over very short distances (1.3 mm) is characterized by marked reduction in the CMAP amplitude with relatively small but significant reductions in CMAP velocity. Only at levels of compression that reduce the CMAP amplitude by more than 80% are nerve conduction velocities reduced by as much as 30–50% and the duration of the CMAP markedly shortened. This reduction in duration could be seen with preferential loss of either more rapidly or more slowly conducting axons. However, the concomitant reduction in nerve conduction velocity suggests that larger axons are preferentially affected. This conclusion is also supported by the fact that larger axons are associated with larger motor units with longer durations so that loss of these larger axons should produce shorter duration CMAP's by this mechanism as well. At first, this might seem to conflict with the data of Battista and Albans [14] who found that acute nerve compression over small lengths of nerve (1 mm) produced injury to slow conducting C fibres before changes in rapidly conducting myelinated axons, while compression over larger lengths (1.2 cm) affected myelinated axons at lower compressions than C fibres. Since the CMAP does not probe the slowly conducting C fibres, the results obtained in this paper refer only to rapidly conducting myelinated axons of alpha motor neurons.

It should be noted that the changes in CMAP amplitude and velocity in this model are very different than those observed during ischemia or nerve stretch [15]. In both of these models there are large increases in latency and reductions in conduction velocity associated with reductions in CMAP amplitude of less than 50%. For example, in the stretch model [15], the conduction velocity decreased 30% when the amplitude was reduced by 50%. In studies that aimed to investigate the effects of ischemia on nerve action potential [16], CMAP [17], and evoked potential [18], it was found that 50% reduction in CMAP amplitude was sufficient to elicit a 20% reduction in nerve conduction velocity, which is again larger than seen in this study. This difference is expected since the region of nerve that might have abnormal conduction velocity is quite substantial in both the ischemia and the stretch models but is tiny in the compression model used in this study. For example, a 50% reduction in conduction velocity across the injured region would cause a 50% reduction in the CMAP conduction velocity if the entire nerve was involved. If only 5% of the length of the nerve were involved (as in our study), there would be only a 5% reduction in the measured conduction velocity. Thus, when monitoring for acute focal compressions, small reductions in the conduction velocity can be clinically significant even though in clinical diagnostic studies of chronic injuries such as carpal tunnel or ulnar neuropathy the criteria for a significant change in conduction velocity is generally a change of at least 10–20%.

Another finding of interest is that higher pressure levels are required to produce significant reductions in the CMAP amplitude when the compression (1000 mmHg) is applied over only 3 minutes than when the compression (200 mmHg) is applied over a longer period of 20 minutes [11]. This difference is consistent with the observation made by Dyck [6] that the changes in fasicular area during compression show a biphasic curve with initial rapid declines over the first few minutes that were attributed to expression of endoneurial fluid followed by slower changes most consistent with the compression of axonal components. This suggests that over short time periods the endoneurial fluid may function as a "shock absorber" that reduces the chance of axonal injury from compression.

From the clinical standpoint, these results are also significant. They provide clinicians with another tool to determine based on relative amplitude and velocity changes whether changes in CMAP are due to ischemia or compression. In addition, it may support the observation of Quinones-Hinojosa [19] that significant changes in the transcranial motor evoked potentials (which bear some similarities to CMAP's recorded after stimulation of motor axons in the cortical and subcortical areas) are associated with shortened durations of the recorded CMAP. In this study, large changes in duration occurred only in the setting of significant compression. However, in the clinical arena, with the many problems involved in obtaining high quality recordings, it important and critical to correlate the changes in multiple variables simultaneously in order to confirm the presence and nature of significant changes in the CMAP. Although, in this study, correlations of changes in multiple variables including the mean CMAP latency and the CMAP latency variance did not help detect statistically significant changes at an earlier point, they would be useful in confirming the presence of significant changes.

It is also clinically significant that the changes in response of a nerve to a low level of compression do, to some extent, predict the response of that same nerve to a larger compression. This suggests that the possibility that variability in recorded CMAP amplitudes during a surgical procedure might be in part a predictor of increased vulnerability to injury.

One caveat to the use of continuous CMAP recording is that, although the CMAP is high in amplitude and thus easy to monitor, changes in the CMAP can be more difficult to interpret than changes the in the nerve action potential. This is primarily a result of the complex time-dependent effects of prolonged stimulation on facilitation and fatigue at the neuromuscular junction and the muscle [20,21]. The complexities of these phenomena are evident in this study. In particular, each experiment was not initiated until the amplitude of the CMAP reached a relatively stable value after the onset of continuous stimulation. This occurred over a period of a few minutes and resulted in a CMAP with an amplitude somewhat less than the maximal value. During low levels of compression, certain nerve fibres become temporarily non-conducting so that some neuromuscular junctions and muscle fibres are not stimulated. Thus, during the recovery period, when function in these fibres return, the neuromuscular junctions have had the opportunity to return toward baseline function and CMAP amplitudes greater than baseline are noted. Of course, at higher levels of compression where nerve fibres are injured to the point where there is no return of function, this phenomenon is not observed.

## Competing interests

The author(s) declare that they have no competing interests.

## Authors' contributions

MMS helped design the compression protocol, developed the data collection software, participated in the compression experiments, the data analysis and writing the manuscript. KB participated in data collection, primarily performed the compression experiments, and participated in the data analysis and checking the manuscript. YMC helped conceive of the study, provided animals for the study, and participated in drafting the manuscript. All authors read and approved the final manuscript.
